# Application of new ARDS guidelines at the bedside

**DOI:** 10.62675/2965-2774.20250171

**Published:** 2025-04-02

**Authors:** Fabia Diniz-Silva, Ary Serpa, Juliana Carvalho Ferreira

**Affiliations:** 1 Universidade de São Paulo Faculdade de Medicina Hospital das Clínicas São Paulo SP Brazil Division of Pulmonology, Instituto do Coração, Hospital das Clínicas, Faculdade de Medicina, Universidade de São Paulo - São Paulo (SP), Brazil.; 2 Monash University School of Public Health and Preventive Medicine Australian and New Zealand Intensive Care Research Centre Melbourne Australia Australian and New Zealand Intensive Care Research Centre (ANZIC-RC), School of Public Health and Preventive Medicine, Monash University - Melbourne, Australia.

Despite advances in the last two decades, acute respiratory distress syndrome (ARDS) has remained a significant challenge in clinical practice, with high mortality rates^(1)^ and a significant long-term impact on survivors.

Since the last guidelines were published in 2017,^(2)^ new studies focusing on different interventions for ARDS have been published, including the use of corticosteroids, extracorporeal membrane oxygenation (ECMO), neuromuscular blocking agents (NMBA) and positive end expiratory pressure (PEEP), leading to revision and update of the guidelines. The updated 2023 guidelines by the European Society of Intensive Care Medicine (ESICM)^(3)^ and the American Thoracic Society (ATS),^(4)^ along with the new global definition of ARDS (2024),^(5)^ provided several evidence-based recommendations for optimizing patient diagnosis and treatment.

The guidelines use the same methodology and make strong recommendations when high-quality clinical trials support the use (or not) of an intervention and conditional recommendations (suggestions) when the evidence is less robust. Both guidelines emphasize the importance of early recognition and diagnosis of ARDS through clinical and imaging assessment to initiate appropriate interventions promptly, thereby reducing the incidence of complications and improving outcomes. The new global definition advances diagnosis by allowing the diagnosis of nonintubated patients receiving oxygen therapy via masks and high-flow nasal catheters. Another advance is the possibility of diagnosing and assessing the severity of hypoxemia using the ratio of pulse oximetry to the fraction of inspired oxygen (SpO_2_/FiO_2_) and assessing lung aeration using ultrasound. These changes in diagnostic criteria significantly impact bedside routines, particularly in resource-limited centers, by enabling early diagnosis and timely initiation of treatment.

According to the two guidelines, the use of low tidal volumes, defined as 4 to 8mL/kg of predicted body weight, is strongly recommended to minimize the risks of barotrauma and volutrauma, thereby reducing the risk of ventilator-induced lung injury. Both guidelines also mention that protective ventilation includes limiting plateau pressure to < 30cmH_2_O although only the ATS guideline includes it in the recommendation statement. Reinforcing this recommendation is important because, despite its widespread recognition, studies have revealed low adherence,^(1,6)^ highlighting challenges in implementing this practice at the bedside and the need for training. Studies conducted during the COVID-19 pandemic^(7,8)^ showed better adherence to the use of protective ventilation and better outcomes, indicating that adequate implementation of this recommendation can have a significant impact on patients. Ventilatory strategies limiting driving pressure are mentioned in both guidelines but remain unresolved due to the lack of large, randomized studies supporting a recommendation, warranting further research.

The two new guidelines make contrasting recommendations in relation to PEEP adjustment. According to the ATS guidelines, high PEEP is recommended for patients with moderate to severe ARDS. The ESICM preferred not to make recommendations regarding the use of PEEP, since studies comparing the strategies of high or low PEEP did not reveal robust results. This lack of consensus may have a negative impact on bedside decision-making regarding PEEP adjustments, since clinicians may choose to use a low or high PEEP/FIO_2_ table depending on the severity of the patient's condition or the clinician's preference. In our opinion, a reasonable strategy would be to preferably use higher PEEP for more severe patients and lower PEEP for the less severe.^(9)^ Both guidelines make a strong recommendation against the use of recruitment maneuvers.

The role of therapies such as prone positioning, ECMO, and the use of NMBAs and corticosteroids are also addressed in the updated guidelines (Table 1).

**Table 1:** Comparison between the American Thoracic Society and European Society of Intensive Care Medicine acute respiratory distress syndrome guidelines

Intervention	ATS 2024	ESICM 2023
Protective ventilation (Vt 4 - 8mL/Kg PBW)	Strong recommendation for use	Strong recommendation for use
Early prone for moderate/severe ARDS	Strong recommendation for use (> 12 hours/day)	Strong recommendation for use (> 16 hours/day)
VV-ECMO for severe ARDS	Conditional recommendation for use	Strong recommendation for use (in an ECMO center)
LRMs	Strong recommendation against use	Strong recommendation against use of prolonged LRMs, weak recommendation against brief LRM
PEEP titration strategy	Conditional recommendation for use of high PEEP *versus* low PEEP in moderate/severe ARDS	No recommendation
Neuromuscular blocking agents	Conditional recommendation for use (48 hours since ARDS onset, severe P/F < 100)	Strong recommendation against the routine use of continuous infusions
Corticosteroids	Conditional recommendation for use	Not addressed
HFOV	Strong recommendation against use	Not addressed
ECCO_2_R	Not addressed	Strong recommendation against use

ATS - American Thoracic Society; ESICM - European Society of Intensive Care Medicine; Vt - tidal volume; PBW - predicted body weight; ARDS - acute respiratory distress syndrome; VV-ECMO - venovenous extracorporeal membrane oxygenation; LRM - lung recruitment maneuver; PEEP - positive end expiratory pressure; P/F - partial oxygen pressure/fraction of inspired oxygen; HFOV - high-frequency oscillatory ventilation; ECCO_2_R - extracorporeal carbon dioxide removal.

According to both guidelines, prone ventilation is recommended for mechanically ventilated patients with moderate to severe ARDS who remain hypoxemic despite conventional ventilatory support, as it has been shown to improve oxygenation and reduce the risk of mortality.^(10)^ Prone positioning requires that the entire team responsible for patient care be properly trained and educated to ensure safety and adequate patient positioning and to avoid displacement of central lines and the endotracheal tube. During the COVID-19 pandemic, prone was widely used,^(7,8)^ which may indicate that the implementation of this strategy has become more widespread. ESICM guidelines suggest the use of awake prone to avoid intubation in patients with acute respiratory failure due to COVID-19, which can be highly valuable for centers with limited resources.

Both guidelines suggest the use of ECMO in patients with severe ARDS with refractory hypoxemia (PaO_2_/FiO_2_ < 80mmHg) despite optimized mechanical ventilation strategies. These patients should be transferred to specialized ECMO centers, and successful implementation of ECMO requires a trained and experienced multidisciplinary team capable of managing potential complications and optimizing respiratory support and may be challenging in low-resource settings, including Brazil, where ECMO is less available in the public health care system.^(11)^

The use of NMBAs is suggested by the ATS guideline in patients with early severe ARDS, while the ESICM guideline does not recommend NMBAs for routine use. NMBAs have been used in mechanically ventilated patients with ARDS to improve patient–ventilator synchrony and gas exchange,^(12,13)^ but the largest and more recent randomized controlled trial did not reveal improved outcomes with their use.^(14)^ In addition, concerns have arisen about the adverse effects of prolonged NMB use, such as muscle weakness and the need for deep sedation. An individualized assessment of the patient's clinical status is important to balance the risks and benefits of NMBAs in ARDS patients. Patients with no improvement in oxygenation, significant patient–ventilator asynchrony despite ventilator adjustments or strong respiratory effort despite sedation may benefit from a short course of NMBAs.^(4,14)^

Corticosteroids are suggested by the ATS guidelines, as their use presents a complex decision-making scenario. A recent trial^(15)^ suggested a decrease in the risk of mortality, a shorter duration of mechanical ventilation, and a shorter hospital stay. This recommendation is contingent on several considerations. Variability in dosage and the timing and duration of treatment between trials have led to uncertainty about optimal treatment protocols. The use of corticosteroids for ARDS may be attractive because of their high availability, low cost, easy administration and potential widespread applicability. However, decisions about the dosage and duration of treatment must be individualized at the bedside, considering specific patient risk profiles. It is important to consider adverse effects, especially in high-risk populations such as immunocompromised individuals or patients with metabolic syndromes.

The new definitions and guidelines provide evidence-based recommendations for the management of patients with ARDS, emphasizing the importance of early diagnosis, the implementation of protective ventilation strategies, and individualized treatment to optimize patient outcomes.
